# Wound-Healing Potential of *Cucurbita moschata* Duchesne Fruit Peel Extract in a Rat Model of Excision Wound Repair

**DOI:** 10.1155/2021/6697174

**Published:** 2021-09-15

**Authors:** Saba Shaygan, Sajad Fakhri, Gholamreza Bahrami, Khodabakhsh Rashidi, Mohammad Hosein Farzaei

**Affiliations:** ^1^Student Research Committee, Kermanshah University of Medical Sciences, Kermanshah, Iran; ^2^Pharmaceutical Sciences Research Center, Health Institute, Kermanshah University of Medical Sciences, Kermanshah, Iran; ^3^Research Center of Oils and Fats, Kermanshah University of Medical Sciences, Kermanshah, Iran

## Abstract

**Materials and Methods:**

Hydroalcoholic extractions of pumpkin fruit peel were obtained and used to prepare two different cold cream-based formulations, namely, 10% and 20% pumpkin peel extracts (PPEs). These formulations, phenytoin cream, and cold cream were topically used once daily for 14 days to compare their wound-healing effects in a rat model of excision wound repair. Wound sizes were monitored at different intervals. Skin tissue samples were subject to H&E staining for histopathological analysis. Blood samples were also taken on day 14 to measure serum levels of nitrite.

**Results:**

Both 10% and 20% PPE formulations resulted in a significant reduction of wound sizes compared to positive and negative controls. Wound closure rate was estimated to be higher in 20% PPE-treated rats. According to histopathological analysis, treatment with 20% PPE improved parameters associated with efficient wound repair, including better regeneration of epidemic layer, higher density of dermis collagen fibers, and lower presence of inflammatory cells. Also, both formulations lowered serum concentrations of nitrite.

**Conclusion:**

Given the obtained data from our study, the hydroalcoholic extract of *Cucurbita moschata* Duchesne fruit peel is proposed to be effective in accelerating the process of excision wound repair partly due to its antioxidant effect in terms of decreasing nitrite concentration.

## 1. Introduction

As the largest organ in human, skin covers the external surface of the body, serving as a protective barrier against invasive pathogens and different types of damages. Skin is a multilayer organ that is directly subject to various harmful microbial, mechanical, thermal, and chemical influences causing skin wounds [1]. Since wound healing and ulcer management impose a substantial health burden, those are regarded as major socioeconomic problems in developing as well as developed countries [2]. Coming in an orderly sequence, wound healing involves a series of events, including homeostasis, inflammation, granulation, and finally scar formation. Upon skin injury, platelets convert fibrinogen to fibrin causing their aggregation and formation of a hemostatic plug which prevents further bleeding. Platelets also secrete several growth factors such as platelet-derived growth factor (PDGF) and transforming growth factor-*β* (TGF-*β*) as well as adhesion molecules able to activate the cells in the surrounding area. During the inflammatory phase, neutrophils infiltrate the injury site and secrete multiple highly active antimicrobial mediators like reactive oxygen species (ROS), cationic peptides, and proteases which play important roles in wound cleaning and prevention of infections [3]. After two days, macrophages come on the scene and trigger or take part in number of processes, including digestion of cell debris and matrix elements [4], release of growth factors and cytokines, and activation of angiogenesis cascade, thereby leading to the entrance of inflammatory and endothelial cells [5]. As part of the granulation tissue formation phase, reepithelialization involves the migration of keratinocytes into the injured area. Besides, fibroblasts invade the wound site and synthesize new extracellular matrix (ECM). Granulation tissue contains proteoglycans, glycosaminoglycans, collagen III, fibronectin, vitronectin, and thrombospondin which promote neovascularization. In response to tremendous angiogenic responses, fibroblasts transform into myofibroblasts which bring together the margins of the wounds to decrease the wound size. Scar tissue formation is considered as the last phase of the wound healing characterized by decreased inflammation and capillary density, apoptosis of myofibroblasts, and replacement of the provisional matrix with a new collagenous matrix [6].

Augmenting evidence highlights the involvement of ROS as critical regulators of several phases of the wound-healing process. ROS are indeed implicated in all wound-healing processes as low levels of ROS are required to cope with invading microorganisms and also for cell survival signaling. However, excessive ROS production or impaired detoxification of ROS causes oxidative damage, which is the major cause of nonhealing chronic wounds. Therefore, a balance of ROS production and scavenging is needed for a timely and efficient wound healing [7].

Traditional-based remedies represent cost-effective, simple, and efficacious alternatives for different types of wounds (burn wounds, ulcers, and infected wounds) by exerting a wide range of therapeutic effects which evoke the process of healing [8]. Herbal-derived compounds are the most extensively used therapeutics for skin lesions. These therapies include the application of herbs, finished herbal products, and herbal preparations which contain biologically active compounds capable of stimulating the healing process. Nowadays, a wide variety of plants, from different parts of the world, are analyzed and applied to treat skin lesions [9–11]. Herbal-based products are prepared as extracts, emulsions, creams, and ointments. Pumpkin, with the scientific name *Cucurbita moschata (C. moschata)*, belongs to the genus *Cucurbita* and the family Cucurbitaceae. Pumpkin is an economically important species with a high production rate. In recent years, it has received a lot of attention due to the health benefits of the compounds derivable from its seeds and fruits [12]. Studies on the chemical and pharmacological features of *C. moschata* extracts derived from its different parts (including seeds, fruits, and stems) have confirmed several biological activities including anticancer [13], antidiabetic [14], antiobesity [15], and hepatoprotective [16] effects. Pumpkin is a nutritionally rich fruit as it possesses *γ*-tocopherol and carotenoid [17]. Also, its peel contains various amino acids, including aspartic acid, glutamic acid, arginine, histidine, glycine, leucine, isoleucine, alanine, methionine, lysine, valine, serine, threonine, tyrosine, and phenylalanine. Wound-healing properties of *C. moschata* Duchesne fruit peel on a rat model of burn wound healing has been previously reported [18]. As ROS and oxidative stress play key roles in the process of wound healing, the critical modulatory activity of *C. moschata* lignan on ROS during the *in vitro* and *in vivo* studies [19] showed related promising results towards skin regeneration. Hence, *C. moschata* seem to be a hopeful candidate in improving wound closure by modulation of oxidative mediators.

The aim of the current study was to evaluate the wound-healing effects of the hydroalcoholic extract of *C. moschata* Duchesne fruit peel in a rat model of excision wound repair.

## 2. Materials and Methods

### 2.1. Chemicals

The chemicals used in this study, including ethanol and glycerin, were from Merck Company (Germany). Ketamine hydrochloride/xylazine hydrochloride solution, hematoxylin and eosin, naphthylethylenediamine dihydrochloride (NEDD), sulphanilamide, and phosphoric acid were from Sigma-Aldrich Chemical Company (USA). Phenytoin cream and cold cream were provided by Behvazan Company (IRAN). As defined, cold cream is a water-in-oil (*W*/*O*) emulsification, usually produced from beeswax and several fragrances. All other chemicals were of highest analytical grade.

### 2.2. Plant Extraction

Pumpkin fruits were purchased from a fruit store located in Kermanshah city, Iran, in October 2019 and were further verified by herbarium experts of the Division of Botany, Department of Biology, Faculty of Science, Razi University, Kermanshah, Iran. The fruits were peeled and air-dried at room temperature. Then, using a mechanical grinder, air-dried peels were pulverized to a coarse powder. Extraction was carried at room temperature in a percolator. In brief, 150 gr of coarse powder was transferred to a percolator and soaked in ethanol/H_2_O (70 : 30 *v*/*v*) as the solvent. Our extraction procedure involved three consecutive 24 h extractions using a new solvent each time. The resultant extracts were then mixed together. The final extract was concentrated using a rotary evaporator at room temperature to obtain a viscous liquid. The resultant concentrated liquid was lyophilized to yield a dry powder extract designated as pumpkin peel extracts (PPEs).

### 2.3. Cream Preparation

Two types of formulations with different concentrations of PPE (10% and 20%) were prepared in cold cream-based formulation as 10% and 20% PPEs, respectively. The cold cream‐based formulations were stored in the refrigerator and were first kept at room temperature for half an hour before being used for wound treatment each time. Cold cream and phenytoin 1% cream were used as negative and positive controls, respectively.

### 2.4. Animals

Male Wistar rats (*n* = 24) weighing between 220 and 250 g were included in this study, divided into 4 groups (*n* = 6). The rats were individually (one rat per cage) housed under standard environmental conditions with 23 ± 1°C temperature and light/dark cycles of 12 h/12 h and fed with normal laboratory food and water *ad libitum*. The experimental study was conducted in accordance with the Guide for the Care and Use of Laboratory Animals and approved by the Committee of Animal Ethics of the Kermanshah University of Medical Sciences (IR.KUMS.REC.1398.400).

### 2.5. Creation of Excision Wound

The rats were anesthetized by intraperitoneal (i.p.) injection of ketamine/xylazine (80/10 mg/kg). The back of the rats was cleaned and shaved. Using a 2.5 cm × 2.5 cm coin, dorsal part was marked, and then, excision wound was created by removing a patch of skin.

### 2.6. Treatment Protocol

The animals were randomly divided into four groups (6 rats in each group): a treatment group receiving 10% PPE cream; a treatment group receiving 20% PPE cream; a positive control group receiving phenytoin cream; and a negative control group receiving cold cream. The day of wound creation was assumed as zero, and the treatment procedure started 24 h after wound creation. Formulations were topically applied to cover the wound surface every 12 h for 14 days. The animals were sacrificed on day 14. On day 14 before sacrifice, blood samples were taken from animals to assess serum levels of nitrite, as a circulating biomarker of oxidative stress.

### 2.7. Wound Contraction Rate

Wound contraction was screened by taking photographs using a digital camera at an equal distance from the wounds and right angle to their surfaces. The captured images were then analyzed by Image J software to measure the wound size (area). The wound closure rate was expressed as the percentage of decrease in the initial wound size (zero-day wound size). The wound closure percentage of each time point was calculated using the following formula: wound closure percentage = (*WA*0 − *WAt*)/*WA*0 × 100, where *WAt* shows the wound area at each time point (days 3, 7, 10, and 14) after treatment and *WA*0 means wound area on day 0.

### 2.8. Histopathological Evaluation

All rats were euthanized with i.p. administration of thiopental sodium [20]. Skin tissue samples from the wound areas were collected on day 14 and preserved in 10% formalin to evaluate the histological changes. Tissue sections underwent hematoxylin/eosin (H&E) staining followed by microscopic imaging under ×100 and ×400 magnifications [21].

### 2.9. Nitrite Assay

In this assay, 100 *µ*l of serum samples were mixed with 50 *µ*l of sulfanilamide (dissolved in H_3_PO_4_ 5%). After incubating at room temperature for 5 min, 50 *µ*l of NEDD solution (0.1% in H_2_O) was added to each supernatant. Optical densities of the samples were measured at 540 nm with an ELISA reader within 30 min. Nitrite concentration was measured using the nitrite standard curve.

### 2.10. Statistical Analysis

All data were expressed as mean values ± standard deviation (S.D.). Data were analyzed by two-way repeated measure analysis of variance (ANOVA) followed by Tukey's post hoc test. A *p* value of less than 0.05 was considered as a statistically significant difference.

## 3. Results

The yield of hydroalcoholic extraction was determined to be 63.12% of the total weight of the dried powder.

### 3.1. Wound Contraction Rate

**Figure 1:**
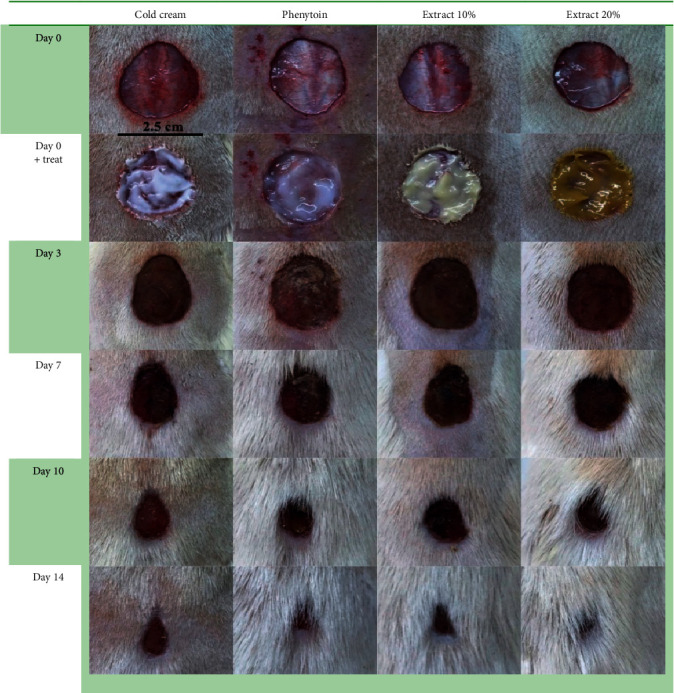
The macroscopic trends of wound healing in control, phenytoin-treated, extract 10%, and extract 20%.

**Figure 2:**
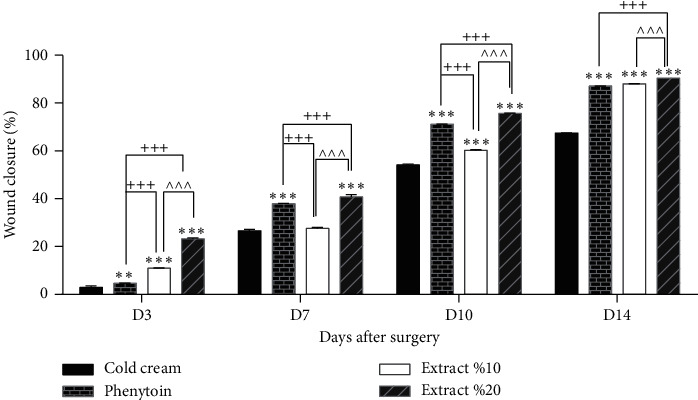
Wound closure percentage of different treated groups on different days after surgery. ^∗∗^*P* < 0.05 and ^∗∗∗^*P* < 0.001, vs. cold cream; ^+++^*p* < 0.001, vs. phenytoin; and ^∧∧∧^*P* < 0.001, vs. extract 10%.

To assess the effects of 10% and 20% extracts on healing of the excision wounds, we measured wound size at different time intervals. Our analysis showed 10% and 20% PPE could significantly decrease the wound size as compared to the negative control group starting from day 3 after surgery. Also, wound-healing activity of 20% PPE cream was significantly higher than 10% PPE cream and phenytoin (positive control) at all the time intervals evaluated. The comparison of wound size between 10% PPE-treated group and phenytoin-treated group revealed that on day 3, 10% PPE did better while its effects on wound contraction rate on days 7 and 10 after surgery were estimated to be weaker than phenytoin. On the final day of evaluation (day 14), however, 10% PPE showed comparable effect on wound closure rate with phenytoin.  Figure 1 shows the macroscopic trends of wound healing, and Figure 2 has indicated associated statistical results.

### 3.2. Histopathological Analysis

**Figure 3:**
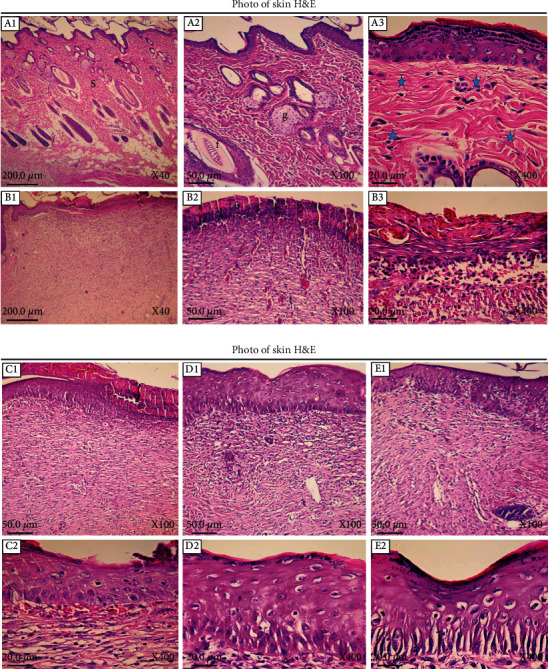
Histopathological evaluation of skin. (A1–A3) Normal healthy skin is a thick tissue (S) anatomically comprising two main segments of the epidermis and dermis. The epidermis consists of a keratinized stratified squamous epithelium (E), while the dermis is a connective tissue with different appendages including hair follicles (f), fat glands (g), and a variety of cells and regular connective tissue arrangements (asterisk). (B1–B3) ×40, ×100, and ×400 magnifications of skin tissue sections of the negative control group, respectively. (C1, C2) ×100 and ×400 images of skin tissue from the 10% PPE-treated group. (D1, D2) ×100 and ×400 magnifications of skin tissue from the 20% PPE-treated group. (E1, E2) ×100 and ×400 images of the phenytoin-treated group.

For histological evaluation of wound area tissue samples on the last day, we used H&E staining.  Figure 3 depicts different magnifications of tissue sections from treatment and control groups. Also, the skin tissue section of a normal skin is presented. In the negative control group, the epidermis was not regenerated in most parts. The dermis showed a detached and irregular structure with disappeared appendages as well as high infiltration of inflammatory cells. Also, scattered collagen bundles were observed as evidenced by a low staining. In 10% PPE-treated group, a similar pattern of tissue damage was visible. However, in 20% PPE- and phenytoin-treated groups (positive control), skin tissue damages were decreased to some extent. Wrinkled epidermis was extensively regenerated, and the dermis exhibited a high density of connective tissue collagen fibers with presence of normal cells.

### 3.3. Nitrite Assay

**Figure 4:**
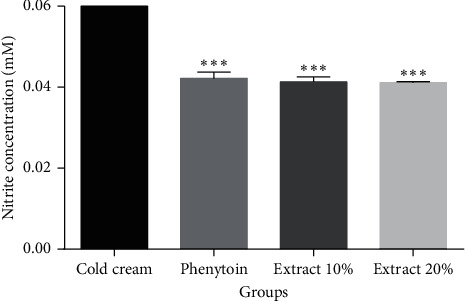
Nitrite concentrations (mm) of serum samples taken on day 14 after wound creation in each group. Cold cream: negative control group; phenytoin: phenytoin-treated group; extract 10%: 10% PPE-treated group; extract 20%: 20% PPE-treated group. ^∗∗∗^*P* < 0.001, vs. cold cream.

To investigate systemic antioxidant activity of PPE, we conducted the nitrite assay on serum samples. Our results were indicative of significantly lower serum concentrations of nitrite in the rats treated with both PPE extracts (10% and 20%) and phenytoin than in the negative control group, indicating a systemic antioxidant mechanism (Figure 4). The changes in the nitrite level are comparable with a normal nitrite level, about 25 *µ*m [22].

## 4. Discussion

The process of wound healing encompasses inflammation, reepithelialization, granulation, and angiogenesis that lead to wound contraction [23]. Wound healing in a shorter time and with less side effects is of the major aims of the current medicine. Various therapeutic strategies have been employed in this regard, including chemical and medicinal remedies and physical approaches such as laser therapy with the main goals of the whole treatments being effective, less toxic, and low-cost healing of the wound within a period as short as possible [2, 8, 24]. Despite the existence of a number of topical preparations in the market, there is still an apparent lack of an appropriate drug. Also, most of the medications or topical preparations available possess antimicrobial activities rather than wound repair effects and are associated with negative side effects and toxicity as is the case regarding silver sulfadiazine on fibroblasts [25]. In this respect, nearly one to three percent of novel medications are suggested to be effective on damaged or normal skin, while about one-third of herbal remedies are used for this purpose [23].

Medicinal plants could exert wound-healing effects due to their wide variety of different constituents and phytochemicals such as alkaloids, flavonoids, fatty acids, terpenoids, saponins, and phenolic compounds that are capable of improving the healing process. Phytochemicals can influence various stages of the wound-healing process through different mechanisms including upregulation of TGF-*β*, vascular endothelial growth factor (VEGF), monocyte chemoattractant protein-1 (MCP-1), and interleukin- (IL-) 1, as well as downregulation of NO and ROS. They also act through enhancing antioxidant power of tissue in the inflammatory phase, increasing matrix metalloproteinase (MMP) and proliferation of endothelial cells during the reepithelialization phase, improving the proliferation of damaged tissue cells in the granulation phase, and boosting angiogenesis by increasing TGF-*β* and VEGF that finally lead to a reduction in wound contraction time [23].

In this study, we showed wound-healing activity of the hydroalcoholic extract of *C. moschata* Duchesne fruit peel in a rat model of excision wound repair. Also, we demonstrated the antioxidant potential of the pumpkin hydroalcoholic extract by nitrite assay. Based on wound closure results, 10% and 20% PPE cream could accelerate the process of wound healing as compared to the negative control. Also, 20% PPE cream exhibited more potent effects than 10% PPE. In a mechanistic point of view, the wound-healing activity of *C. moschata* peel extract could be attributed to its high mucilage content and presence of different constituents such as flavonoids and phenolic compounds that are able to accelerate wound healing as well as its antioxidant power [18]. The most relevant study to our study is that of Bahramsoltani et al. investigating burn wound-healing activity of the hydroalcoholic extract of *C. moschata* Duchesne fruit peel. They elucidated beneficial effects of pumpkin peel extract (PPE) on a rat model of burn wound healing [18]. As shown by their study, PPE has a high mucilage content. Accumulating evidence suggests that a moist environment supports wound healing through several mechanisms, including stimulation of reepithelialization, angiogenesis, keratinocyte migration, and induction of hypoxia inducible factor-1 (HIF-1) that results in the production of endogenous stimulants of wound healing [26]. Thus, high mucilage content can partly account for the ameliorating effects of our PPE on excision wounds. Another group of phytochemicals accounting for the beneficial effects of our PPE includes flavonoids and phenolic compounds as the ethanolic extract of *C. moschata* Duchesne has been shown to contain these compounds [27]. There are several studies on the wound-healing activities of different parts of pumpkin including the study by Ben Halima and colleagues conducted on pumpkin (*Cucurbita peop* L.) seed oil [28]. Dried seed of pumpkin contains oil from 40% to 50% which is also shown to be rich in polyunsaturated fatty acids [29]. In their *in vivo* study, pumpkin seed oil did significantly better in healing the skin wounds than the reference group receiving Cicaflora cream (an emulsion containing 10% of *Mimosa tenuiflora* extract). The group treated with pumpkin seed oil experienced full reepithelialization [28].

From the mechanistic point of view, regulation of ROS levels in wounded skin is necessary for an efficient wound repair. ROS, on the one hand, can contribute to the clearance of invading bacteria and are also key regulators of numerous intracellular signaling pathways at physiological concentrations. On the other hand, augmented levels of ROS can lead to cell damage and delay the process of wound repair [30]. In this regard, NO (as a type of ROS) also plays a dual role. Its high concentration can cause oxidative damage and hinder wound repair while it is also involved in cell proliferation, collagen formation, and wound contraction [31]. Our study showed that PPE can decrease serum nitrite levels, reflecting its systemic antioxidant activity to precipitate wound repair. In line with our results, Bahramsoltani et al. reported the antioxidant potential of PPE in terms of decreasing biomarkers of tissue oxidative stress. Based on their results, treatment with 10% and 20% PPE significantly lowered lipid peroxidation in skin tissues. Also, 20% PPE could augment total antioxidant power (TAP) of the damaged tissues. Additionally, *in vitro* evaluations including 1,1-diphenyl-2-picrylhydrazyl (DPPH) radical scavenging and ferric-reducing antioxidant power (FRAP) tests were also indicative of antioxidant properties of the extract [18]. In another study, the pumpkin extract was shown to upregulate the activities of antioxidant enzymes such as glutathione peroxidase (GSH-Px) and superoxide dismutase (SOD) and also repressed lipid peroxidation in mice [32]. In accordance with these findings, it has been demonstrated that systemic administration of the pumpkin extract reduces malondialdehyde (MDA) and upregulates liver GSH-Px and SOD [33]. Also, investigations have confirmed antioxidant properties of pumpkin seed oil and fruit extract [34].

Nowadays, the fabrication and administration of novel delivery systems have also paved the road in wound healing. In this line, using the encapsulation of epidermal growth factor in gelatin-alginate coacervates could significantly reduce the wound area by suppressing inflammatory cytokines (e.g., IL-1, IL-6, and TNF-*α*) [35].

Taken together, our study showed wound-healing activity of *C. moschata* Duchesne fruit peel extract in a rat model of excision wound repair. Also, as a mechanism of action, we showed the antioxidant capability of the hydroalcoholic extract of *C. moschata* Duchesne as reflected by reduction in nitrite levels in serum samples of the treated rats. Our data shed more light on the wound-healing activity of the peel extract of pumpkin. However, further supportive experimental and clinical investigations are required to approve the peel extract of this kitchen fruit as a wound-healing remedy.

## Data Availability

The datasets used in the present study are available from the corresponding authors on reasonable request.
